# Enhancement of mouse sperm motility by trophinin-binding peptide

**DOI:** 10.1186/1477-7827-10-101

**Published:** 2012-11-29

**Authors:** Seong Kyu Park, Jiwon Yoon, Ling Wang, Toshiaki K Shibata, Khatereh Motamedchaboki, Kyung Jun Shim, Mun Seog Chang, Seung Ho Lee, Naoaki Tamura, Shingo Hatakeyama, Daita Nadano, Kazuhiro Sugihara, Michiko N Fukuda

**Affiliations:** 1 Tumor Microenvironment Program, Cancer Center, Sanford-Burnham Medical Research Institute, 10901 North Torrey Pines Rd, La Jolla, CA 92037, USA; 2 Department of Prescriptionology, College of Oriental Medicine, Kyung Hee University, Seoul, 130-701, Republic of Korea; 3 Department of Gynecology and Obstetrics, Hamamatsu University School of Medicine, Hamamatsu City, Shizuoka, 431-3192, Japan; 4 Department of Urology, Hirosaki University School of Medicine, Hirosaki, Aomori, 036-8562, Japan; 5 Department of Applied Molecular Biosciences, Graduate School of Bioagricultural Sciences, Nagoya University, Chikusa, Nagoya, 464-8601, Japan

**Keywords:** Microtubule, Flagella, Dynein, ATP, Mutant, Gene knockout

## Abstract

**Background:**

Trophinin is an intrinsic membrane protein that forms a complex in the cytoplasm with bystin and tastin, linking it microtubule-associated motor dynein (ATPase) in some cell types. Previously, we found that human sperm tails contain trophinin, bystin and tastin proteins, and that trophinin-binding GWRQ (glycine, tryptophan, arginine, glutamine) peptide enhanced motility of human sperm.

**Methods:**

Immunohistochemistry was employed to determine trophinin protein in mouse spermatozoa from wild type mouse, by using spermatozoa from trophinin null mutant mice as a negative control. Multivalent 8-branched GWRQ (glycine, tryptophan, arginine, glutamine) peptide or GWRQ-MAPS, was chemically synthesized, purified by HPLC and its structure was confirmed by MALDI-TOF mass spectrometry. Effect of GWRQ-MAPS on mouse spermatozoa from wild type and trophinin null mutant was assessed by a computer-assisted semen analyzer (CASA).

**Results:**

Anti-trophinin antibody stained the principal (central) piece of the tail of wild type mouse sperm, whereas the antibody showed no staining on trophinin null sperm. Phage particles displaying GWRQ bound to the principal piece of sperm tail from wild type but not trophinin null mice. GWRQ-MAPS enhanced motility of spermatozoa from wild type but not trophinin null mice. CASA showed that GWRQ-MAPS enhanced both progressive motility and rapid motility in wild type mouse sperm.

**Conclusions:**

Present study established the expression of trophinin in the mouse sperm tail and trophinin-dependent effect of GWRQ-MAPS on sperm motility. GWRQ causes a significant increase in sperm motility.

## Background

Trophinin was identified as an apical cell adhesion molecule mediating adhesion between trophoblastic and endometrial epithelial cells at the time of human embryo implantation [1,2]. Trophinin is a homophilic cell adhesion molecule; trophinin expressed on trophectoderm cells of blastocyst stage embryos binds to trophinin expressed on maternal endometrial epithelial cells [1]. Trophinin exhibits an N-terminal cytoplasmic tail, followed by a membrane-spanning domain and an extracellular domain composed of tandem decapeptide repeats. Trophinin gene knockout mouse reproduce normally, demonstrating that trophinin does not function in mouse embryo implantation [3]. By contrast, several lines of evidence suggest that trophinin’s function in embryo implantation is unique to humans [4-8].

The trophinin gene is found only in mammals. In both human and mouse, it gives rise to two distinct proteins, magphinin and trophinin [9,10]. Magphinin proteins are expressed in the male and female germ cells. It was suggested that magphinins regulate proliferation of germ cells during gametogenesis [9]. A gene expression database analysis ( http://www.ncbi.nih.gov/UniGene) indicates that magphinin/trophinin transcripts are expressed in the brain, testis, thymus, kidney, and parathyroid gland in mouse and human, suggesting yet undefined role of trophinin gene products in cells of these organs. Immunohistochemistry using trophinin-specific antibody detects strong signals for trophinin protein in distinct cell types of the germinal zone in adult rat brain [11] and in mature spermatozoa in the mouse testis [9]. However, trophinin gene knockout mice do not show apparent neurological defects or male infertility [3], leaving trophinin’s role in neuronal or spermatogenic cells elusive.

Previously we screened a peptide-display phage library and identified a trophinin-binding peptide GWRQ [12]. GWRQ peptide was capable of transmitting cell surface signals promoting proliferation and invasion to the cytoplasm of human trophoblastic cells [12,13] as well as apoptotic signals in human endometrial epithelial cells [14,15]. We also found that GWRQ peptide bound to human sperm tail and promoted sperm motility [16]. Since it is known that trophinin-associated cytoplasmic protein, tastin, directly binds dynein ATPase [17], we hypothesized that GWRQ binding to the trophinin extracellular domain releases tastin from trophinin and into the cytoplasm, enabling it to activate dynein ATPase. Here, we determined the activity of GWRQ peptide on mouse sperm. To do so, we asked whether GWRQ peptide promotes sperm tail motility in a trophinin-dependent manner by taking advantage of the availability of trophinin null mouse [3].

## Methods

### Animals and animal experiments

The trophinin gene knockout mouse was generated as reported previously [3]. Mice were back-crossed to the C57BL/6 strain for more than 9 generations and maintained by breeding between Tro+/− females and wild-type C57BL/6 males. Genotyping was performed by PCR (polymerase chain reaction) using genomic DNA isolated from tail biopsy of 3-week-old pups, as described [3]. Protocols for use of mice in this study were approved by an institutional animal research committee at the Sanford-Burnham Medical Research Institute.

### Peptides

Multivalent 8-branched GWRQ peptide, known as GWRQ-MAPS (multivalent antigenic peptides), was described previously [12,16]. Crude GWRQ-MAPS synthesized by GenScript was fractionated twice by gel filtration using a Sephadex G-25 column (1.5 × 40 cm) equilibrated with 30% acetonitrile in water (see Additional file 1: Figure S1A). GWRQ-MAPS which was eluted as high molecular weight fraction was purified by a C18 reverse-phase HPLC (high performance liquid chromatography) column (10 x 150 mm) in HPLC (Shimadzu LC-10 AD) by elution in a linear gradient from 20% to 40% acetonitrile in water containing 0.01% trifluoro acetic acid over 30 minutes at a flow rate of 2.5 ml/min. Purity of GWRQ-MAPS was validated by HPLC (see Additional file 1: Figure S1B). The structure of HPLC-purified GWRQ-MAPS was confirmed by Matrix-Assisted Laser Desorption Ionization (MALDI)-TOF/TOF mass spectrometry on a Brucker Daltonics Autoflex II TOF/TOF mass spectrometer (Bruker Daltonics Inc.,Billerica, MA) (see Additional file 1: Figure S1C).

### Antibodies

Polyclonal rabbit anti-mouse trophinin antibody was raised by immunizing rabbits with synthetic peptide CTSTDFSGGLNHNADFN, which represents T880 to N895 of full-length mouse trophinin protein [3], and an N-terminal C was added for conjugation to carrier protein. Proteins were precipitated from antisera using 50% saturated ammonium sulfate. Precipitates were dialyzed against PBS, and solubilized proteins were applied to an affinity chromatography column of the synthetic peptide described above conjugated to Sulfo-link agarose beads (Pierce). Peptide column-bound antibody was eluted with 0.2 M glycine-HCl buffer, pH 2.4, neutralized with 1/5 volume of 1 M Tris–HCl buffer, pH 8.0, and stored at −80°C in the presence of 6% trehalose.

### Immunohistochemistry

Mouse testis tissues from wild-type and trophinin null male mice were fixed with 4% paraformaldehyde in PBS (phosphate buffered saline) and embedded in O. C. T. compound (Tissue-Tek). Frozen sections were placed on glass slides, air-dried, and post-fixed with paraformaldehyde. Mouse spermatozoa were released from ductus deferens into PBS. Sperm cells were smeared on the glass slide glass, air-dried, and fixed with 4% paraformaldehyde in PBS. Slides containing sperm were then treated with 0.01 N HCl at 90°C for 1 hour to remove sialic acids or glycan layers covering the sperm tail, as described [16]. Sperm or tissue sections were then treated with 0.3% hydrogen peroxide in methanol at room temperature for 30 min. After blocking tissue with 10% normal goat serum, sections were reacted with affinity-purified rabbit anti-trophinin antibody described above, followed by reaction with biotinylated anti-rabbit IgG antibody (Vector), and peroxidase avidin. Negative controls for immunostaining were performed without primary antibody. The color reaction was performed using AEC solution (Invitrogen). Counter-staining was performed with hematoxylin (Vector).

### Peptide-displaying phage

GWRQ-peptide displaying filamentous fd phage was isolated as described previously [12]. Phage was recovered from the culture medium of K91Kan bacterial cells infected by the GWRQ phage, and precipitated by 6% polyethylene glycol and 0.32 M NaCl. After dialysis against PBS, phage was stored at −80°C. Sperm cells smeared on a slide glass were treated with mild acid hydrolysis as described above. Phage solution suspended in PBS containing 1 mM EDTA (ethylene diamine tetraacetate) was overlayed on the slide, incubated at room temperature for 5 min, and washed with PBS. Sperm cells were then fixed with 4% paraformaldehyde at room temperature for 15 min, and treated with 0.3% hydrogen peroxide in methanol at room temperature for 30 min. Phage bound to the sperm cells was detected by rabbit anti-phage fd antibody (Sigma), biotinylated anti-rabbit IgG antibody, and peroxidase avidin. The color reaction was performed using AEC. Counter-staining was with hematoxylin.

### Sperm motility assay

Mature spermatozoa released from ductus deferens of were collected into 2 ml of M199 medium (Invitrogen, Carlsbad, CA) supplemented with 0.5% bovine serum albumin. Adequate dispersion was determined visually, and an aliquot was diluted in the same medium. An aliquot of the sperm suspension, without fractionation into faster and slower swimming populations, was divided into two groups. Sperm cells in 200 μl sperm-washing medium was mixed with an equal volume of medium containing GWRQ-MAPS at final concentration 50 μg/ml. Sperm numbers and motility were evaluated after incubation at 37°C for 30 min. A sperm aliquot was placed on a pre-warmed 100 μm deep counting chamber slide (Hamilton Thorne Research, Danvers, MA) and analyzed using a computer-assisted semen analysis (CASA) (Leja, Nieuw-Vennet, Netherlands). Sperm from 4 wild type and 4 trophinin null males (8–14 weeks old) were subjected to CASA. At least 90 sperm cells/field and total 10 fields/mouse were assessed by CASA for each treatment group. Parameters assessed were those recommended by the manufacturer for mouse sperm, and they are: total motility, progressive motility, rapid motility, VAP, average path velocity (μm/sec); VSL, straight line velocity (μm/sec); VCL, continuous line velocity (μm/sec); ALH, amplitude of lateral head displacement (μm); BCF, beat cross frequency (Hz); STR, straightness (ratio of VSL/VAP); LIN, linearity (ratio of VSL/VCL).

### Statistical analysis

Statistical analysis using Wilcoxon signed rank test and Student’s *t*-test was performed using the Graph Pad Prism program. A *P* value less than 0.05 was considered statistically significant.

## Results

### Localization of trophinin protein in mouse spermatozoa

**Figure 1:**
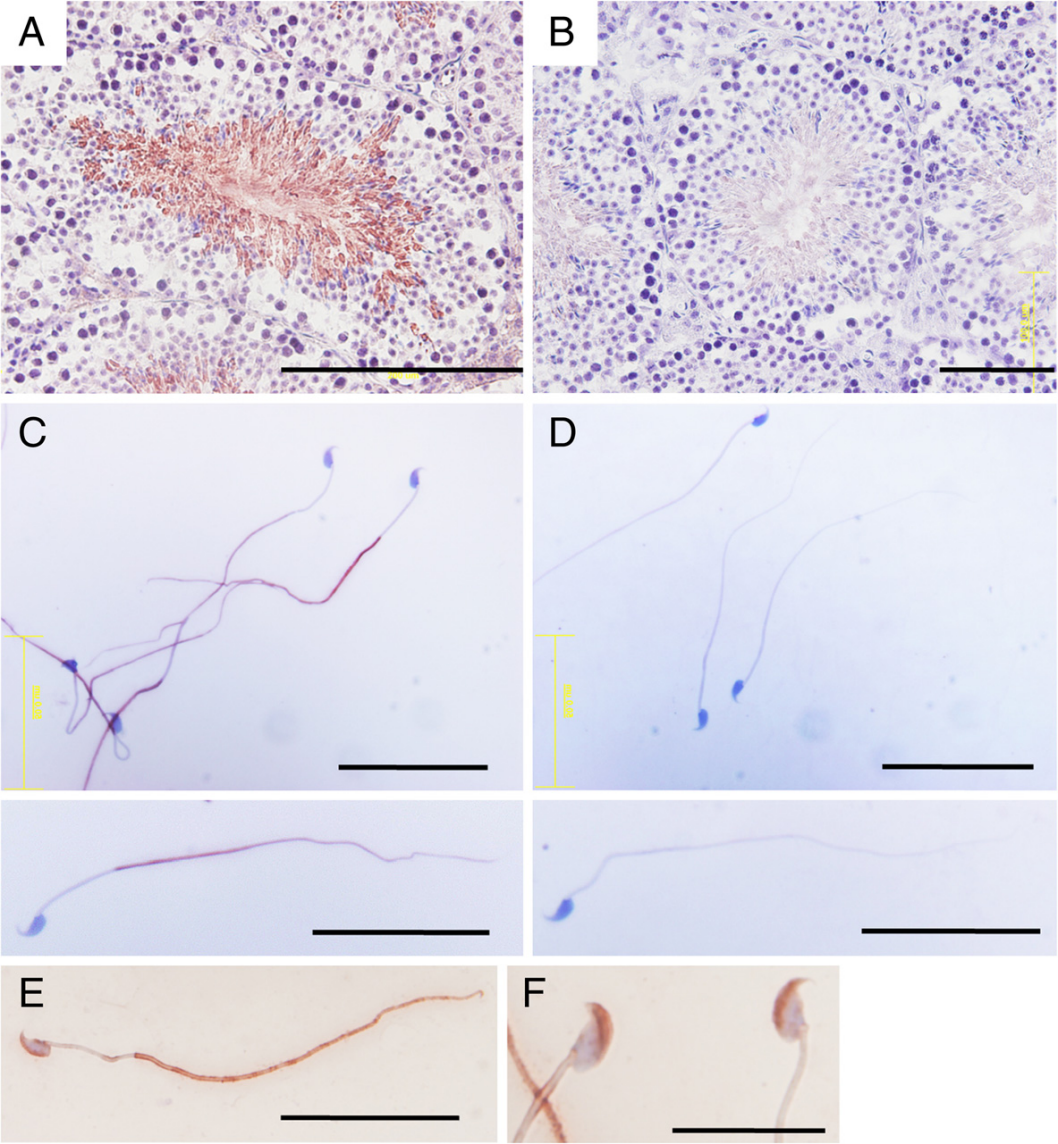
**Immunohistochemistry of mouse testis sections and mature spermatozoa using anti**-**trophinin antibody.** Frozen sections of mouse testes each from wild-type (**A**) and trophinin null (**B**) mice were stained using rabbit anti-trophinin antibody. Matured spermatozoa released from ductus deferens from wild-type (**C**) and trophinin null (**D**), and immature spermatozoa released from Caput epididymis (**E** and **F**). Scale bars 200 μm (**A**), 100 μm (**B**), 20 μm (C-E), and 10 μm (**F**).

To determine where trophinin protein was localized in mouse sperm, we reacted frozen tissue sections of testes from wild-type and trophinin null mice with rabbit anti-trophinin antibody (Figure 1A, B). The antibody stained mature sperm cells in wild-type testis, consistent with previous reports [9]. The antibody did not stain sections from trophinin null mice, confirming its specificity.

Previously we found that monoclonal anti-trophinin antibody or GWRQ-displaying phage particles did not bind to human sperm tails due to heavy glycosylation; however, after mild acid treatment, both antibody and phage bound to sperm tails [16]. In the mouse sperm experiments, mature mouse spermatozoa released from ductus deferens also were not stained by rabbit anti-trophinin antibody described above (data not shown). However, after mild acid treatment, these spermatozoa from wild type mouse were stained by anti-trophinin antibody (Figure 1C). The antibody stained the tail principal piece, but not the head, the tail midpiece or the tail end piece. Spermatozoa from trophinin null mice showed no signals (Figure 1D).

Trophinin staining patterns in wild-type mouse spermatozoa differed from those we previously reported in human sperm cells, which show an intermittent, stripe-like pattern along the anterior-posterior axis of the sperm tail [16]. In human sperm, trophinin is also detected in the neck and in the tail midpiece [16]. Since the staining pattern seen in mouse testis suggested the existence of trophinin protein in the sperm head (Figure 1A), we asked if spermatozoa at early stages of maturation express trophinin in the head. Spermatozoa released from Caput epididymis showed trophinin staining in the head (Figure 1EF). These results suggest that while at early maturation stages, trophinin proteins are widely distributed in spermatozoa, trophinin is restricted to the principal piece in fully mature spermatozoa.

### Binding of GWRQ peptide to mature spermatozoa

**Figure 2:**
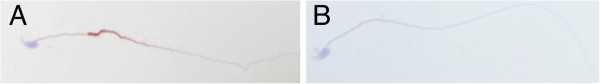
**GWRQ phage binding to mouse sperm visualized by immunohistochemistry using anti**-**phage antibody.** Spermatozoa were treated with mild acid hydrolysis to remove glycans followed by incubation with GWRQ-peptide displaying phage. Phage binding to sperm cells was visualized by immunohistochemistry using anti-phage antibody. **A**) Wild-type sperm. **B**) Trophinin null sperm.

To determine if trophinin-binding peptide GWRQ binds to the mouse sperm tail, GWRQ phage particles were overlayed onto fixed mature spermatozoa on slides and phage binding to spermatozoa was visualized by immunostaining with an anti-phage antibody. This analysis showed positive phage binding to wild-type sperm but no binding to trophinin null sperm (Figure 2A,B). GWRQ phage bound to the principal piece where trophinin proteins are localized. These results strongly suggest that GWRQ peptide binds to mouse sperm tails in a manner dependent on trophinin protein.

### Effect of GWRQ-MAPS on sperm motility

**Figure 3:**
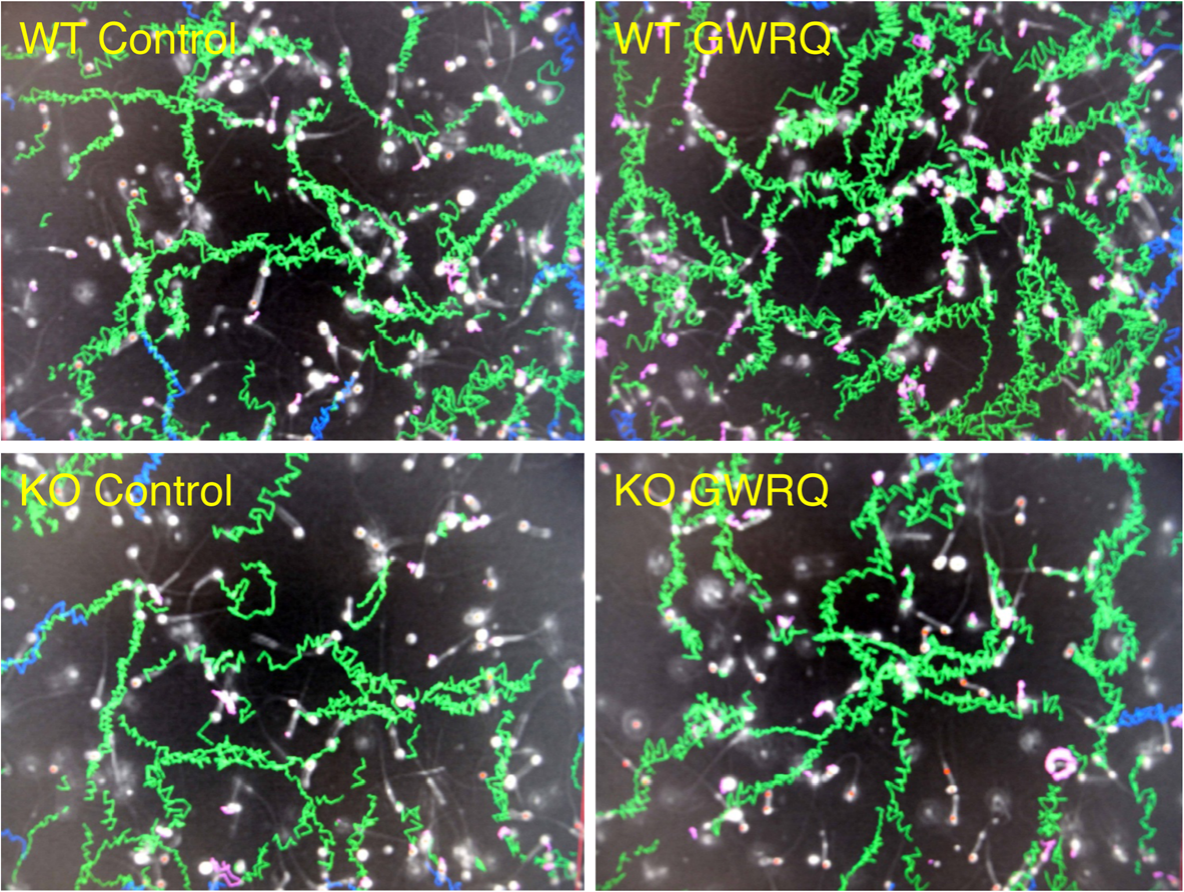
**Sperm tracks generated by CASA.** Green tracks illustrate movement of individual sperm, and red dots indicate immotile or dead sperm. **A**) Wild-type mouse spermatozoa without (Control) or with GWRQ peptide. **B**) Trophinin null spermatozoa with or without GWRQ-MAPS.

The effect of GWRQ on mouse sperm motility was determined using synthetic and highly purified 8-branched GWRQ peptide, or GWRQ-MAPS (see Additional file 1: Figure S1). Visual assessment of GWRQ-treated mouse sperm suggested that they were hyperactive and swam randomly compared to movement exhibited by control sperm without peptide (Figure 3). Total motility of spermatozoa was enhanced in a manner dependent on GWRQ-MAPS, showing the optimum concentration at 50 μg/ml (see Additional file 2: Figure S2). Thus, in subsequent experiments, we used GWRQ-MAPS at 50 μg/ml.

**Figure 4:**
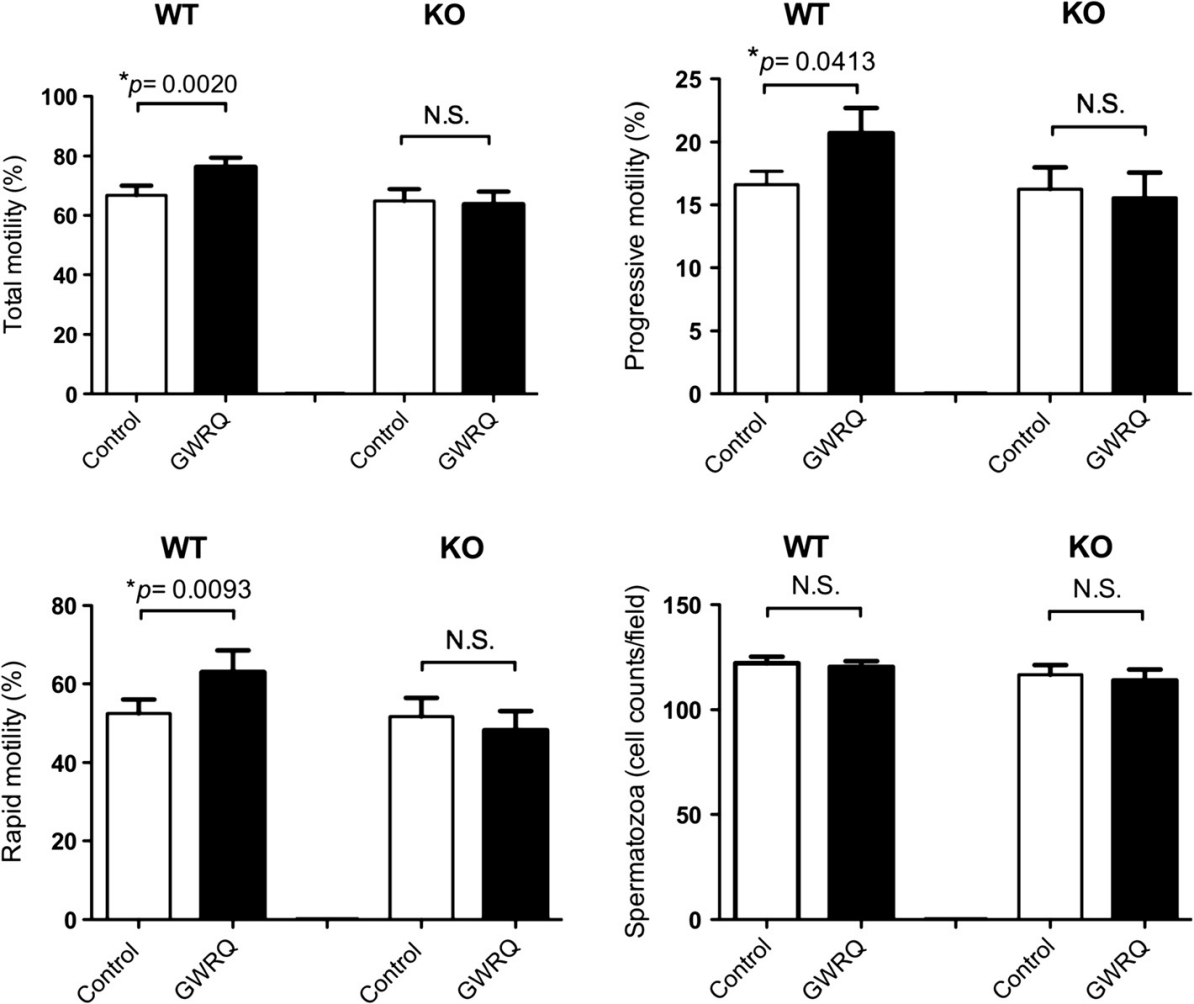
**Effect of GWRQ on motility parameters of wild**-**type and trophinin null mutant mice.** Sperm were collected and incubated in sperm washing medium. CASA was performed after incubation for 30 min. Graphs represent the means +/− SEM of 3 independent experiments. **A**) Numbers of spermatozoa. **B**) Total sperm motility. **C**) Progressive motility. **D**) Rapid motility. Asterisks indicate significant differences between control and GWRQ groups (P < 0.05, Wilcoxon signed rank test).

For quantitative analysis of motility changes, CASA was performed after exposing sperm to GWRQ-MAPS at 37°C for 30 min. When wild-type spermatozoa were incubated in medium containing GWRQ-MAPS, total motility and progressive motility were enhanced (Figure 4), accompanied by enhancement of VAP, VSL, and VCH parameters (see Additional file 3: Figure S3). Differences in motility parameters were significant between sperm samples with or without GWRQ-MAPS. By contrast, the peptide had no effect on trophinin null spermatozoa.

## Discussion

The present study based on comparisons between wild type and trophinin null spermatozoa showed that trophinin proteins are expressed on mouse sperm tails and that the trophinin-binding peptide GWRQ-MAPS can promote mouse sperm motility. We previously observed similar motility-enhancing activity of GWRQ-MAPS on human sperm [16]. Therefore we anticipate that GWRQ-MAPS can promote sperm motility in numerous mammals, which could be addressed in future studies.

Although trophinin null male mice did not exhibit infertility [3], when we isolated sperm from 4–5 month old trophinin null male mice from twice back-crossed or the second generation of the C57BL/6 strain, we observed that sperm were often immotile (LW and MNF, unpublished observations). However, in trophinin null male mice from subsequent back-crosses, trophinin nulls showed no apparent spermatozoa motility defect, suggesting that the sperm motility defect seen in trophinin null males is dependent on genetic background. It is possible that defects in tail motility associated with trophinin null male mice are more pronounced in the 129/SvJ background in which the trophinin null ES line was generated [3] than in a C57BL/6 background.

Here, we found using CASA that GWRQ-MAPS peptide activated tail motility of wild-type mouse spermatozoa (Figure 3 and 4), confirming our previous study of human sperm showing that GWRQ-MAPS enhanced sperm motility [16].

Sperm tail motility requires movement produced by the energy of the microtubule-associated ATPase, dynein, in an evolutionally conserved manner [18-21]. Trophinin associates with bystin and tastin in trophoblastic cells [1,2]. We previously found that dynein binds to tastin [17] and that tastin binds to trophinin through bystin in the cytoplasm [1,17,22,23]. In human sperm tail, trophinin, bystin, and tastin proteins are apparently co-expressed [16]. It is likely that the trophinin N-terminal cytoplasmic domain associates with dynein in the mouse sperm tail, providing a potential mechanism for GWRQ-mediated hyperactivation of sperm tails.

Sperm motility requires high ATP levels to support coordinated movement of the central axoneme and surrounding flagellar structures [24]. ATP is generated mainly by oxidative phosphorylation in mitochondria located in the midpiece. However, ATP generated there must be supplied to the principal piece of the tail, requiring energy [19]. Recent findings in mice deficient in the sperm-specific glycolytic enzyme, glyceraldehyde 3-phosphate dehydrogenase-S (GAPDS), suggest that ATP generated by glycolysis at the principal piece is essential for sperm motility [25]. GAPDS localizes to the principal piece of the mouse sperm tail [25], where trophinin is localized (Figure 1B). GAPDS and other germ cell-specific glycolytic enzymes may be critical to ensure a sufficient localized supply of ATP along the length of the sperm flagellum for sperm motility. The close proximity of trophinin and GAPDS suggests that the trophinin/bystin/tastin complex may arrest dynein ATPase in a manner similar to that we reported for ErbB4 in trophoblastic cells [12,13]. In silent trophectoderm cells, trophinin arrests ErbB4 through bystin in the cytoplasm and therefore HB-EGF (heparin-binding EGF-like growth factor) bound to ErbB4 does not promote autophosphorylation of the ErbB4 cytoplasmic domain. When GWRQ binds the trophinin extracellular domain, bystin is dissociated from ErbB4, allowing ErbB4 to undergo autophosphorylation [6]. Since it is known that tastin directly binds dynein ATPase [21], we hypothesize that GWRQ binding to the trophinin extracellular domain releases tastin from trophinin in the cytoplasm, enabling it to activate dynein ATPase. We speculate that a trophinin, bystin and tastin complex arrests dynein, so that dynein cannot hydrolyze ATPs locally supplied by glycolysis. Thus trophinin may play a role in conserving ATP in the principal piece during long distance travel toward the oocyte, where sperm need to move vigorously to achieve fertilization.

## Conclusions

The present study established the presence and location of trophinin protein in mouse sperm by immunohistochemistry using wild type and trophinin null spermatozoa. Effect of trophinin-binding GWRQ peptide was confirmed to be trophinin-dependent by analyzing sperm motility by CASA using wild type and trophinin null spermatozoa.

## Competing interests

The authors declare that they have no competing interests.

## Authors’ contributions

SKP carried out sperm motility assays and analyzed the data. JY carried out the sperm motility assay and immunohistochemistry. LW prepared sperm cells from wild type and mutant mice. TKS participated sperm motility assay. KM analyzed GWRQ-MAPS peptide by mass spectrometry. KJS purified GWRQ-MAPDS and genotyped trophinin gene knockout mouse. MSC and SHL analyzed the data. NT participated sperm motility assay. SH participated the data analysis. DN generated trophinin gene knockout mouse line. KS and MNF design the study, analyzed the data and drafted the manuscript. All authors read and approved the final manuscript.

## Supplementary Material

Additional file 1 **Figure S1.** Purification and validation of synthetic GWRQ-MAPS peptide. Gel filtration of crude GWRQ-MAPS (A), HPLC purification (B), and mass spectrometry analysis of purified GWRQ-MAPS (C). HPLC showed purity of GWRQ-MAPS more than 99%. Signal at m/z 5120.835 (major) is theoretical mass for GWRQ-MAPS, 5135.886, minus 15. Apparent dimer (m/z 10244.897) and trimer (m/z 15335.252) were formed during storage after HPLC purification. Click here for file

Additional file 2 **Figure S2.** Dose-dependency of GWRQ-MAPS on motility of wild type mouse sperm. Fresh spermatozoa from three C57BL/ 6 mice were analyzed by CASA. Each bar represents the result obtained by ten randomly selected fields each containing >100 spermatozoa. Asterisks show statistical significance (p<0.05). Click here for file

Additional file 3 **Figure S3.** CASA parameters. Data were collected by analyzing fresh sperm frosignificant (pm wild type (n=10) and trophinin null (n=3) mice. Asterisks represent statistical < 0.05) by unpaired two-tailed t-test. N. S., not significant. Click here for file
