# Supplementary figures and images for: Enhancement of mouse sperm motility by trophinin-binding peptide
Source: Reprod Biol Endocrinol. 2012 Nov 29;10:101. doi: 10.1186/1477-7827-10-101 (PMC3551822; doi:10.1186/1477-7827-10-101)

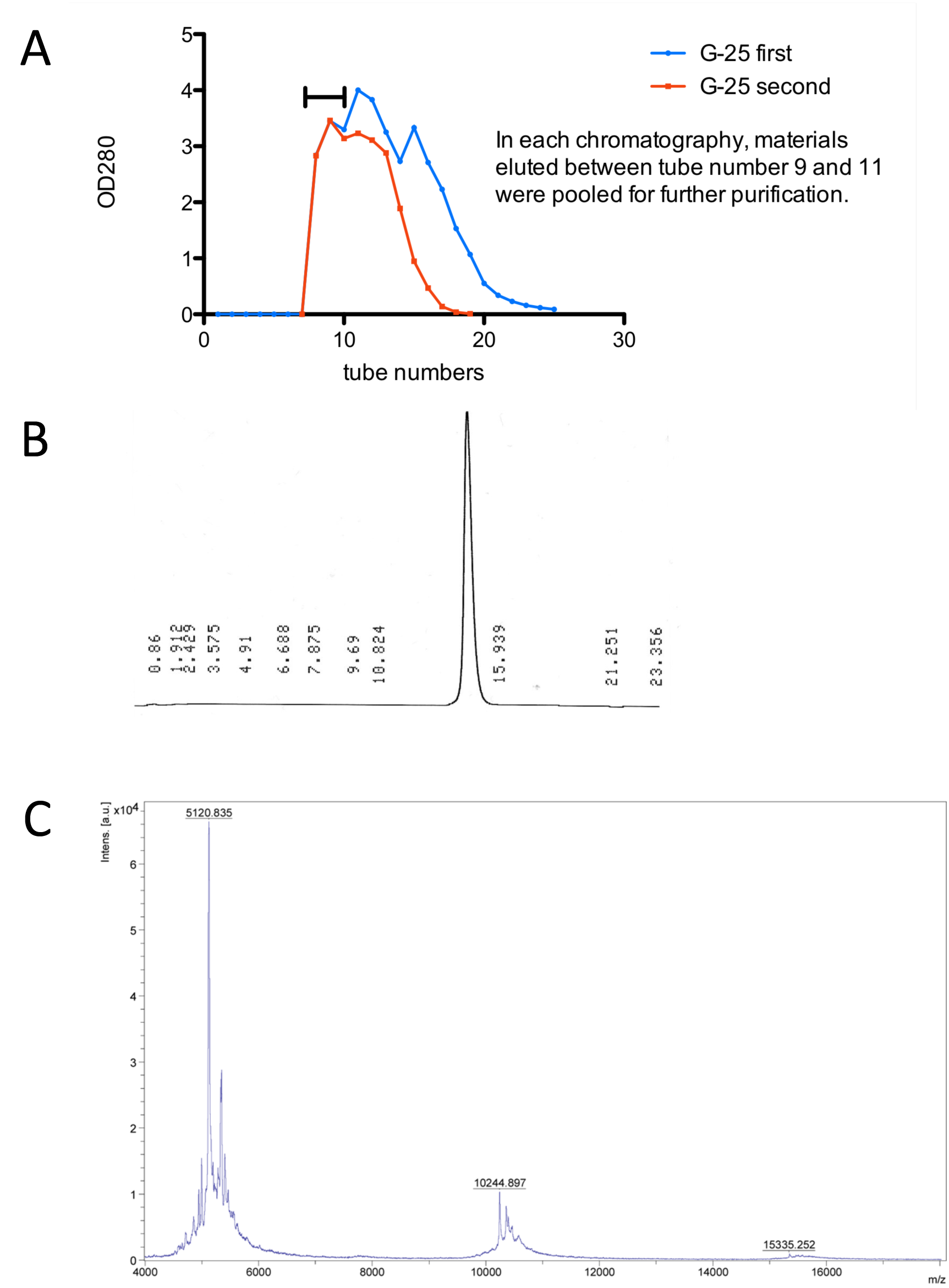

Supplement: Additional file 1 — Figure S1. Purification and validation of synthetic GWRQ-MAPS peptide. Gel filtration of crude GWRQ-MAPS (A), HPLC purification (B), and mass spectrometry analysis of purified GWRQ-MAPS (C). HPLC showed purity of GWRQ-MAPS more than 99%. Signal at m/z 5120.835 (major) is theoretical mass for GWRQ-MAPS, 5135.886, minus 15. Apparent dimer (m/z 10244.897) and trimer (m/z 15335.252) were formed during storage after HPLC purification. [file 1477-7827-10-101-S1.tiff]

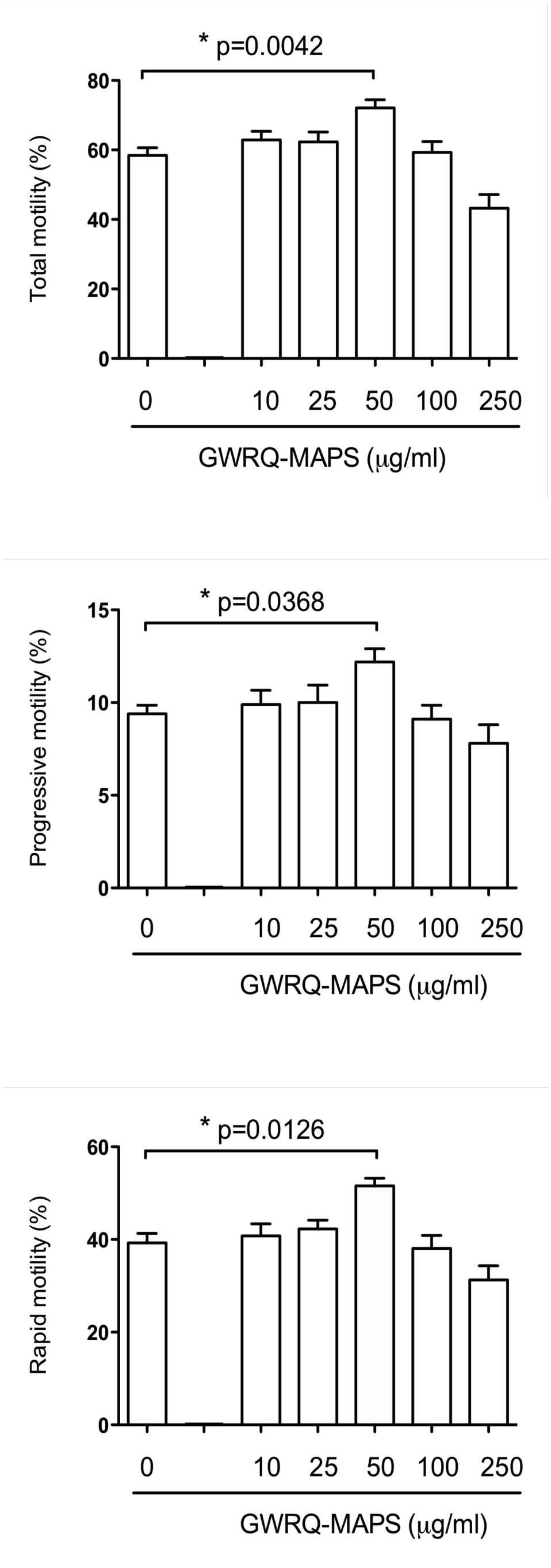

Supplement: Additional file 2 — Figure S2. Dose-dependency of GWRQ-MAPS on motility of wild type mouse sperm. Fresh spermatozoa from three C57BL/ 6 mice were analyzed by CASA. Each bar represents the result obtained by ten randomly selected fields each containing >100 spermatozoa. Asterisks show statistical significance (p<0.05). [file 1477-7827-10-101-S2.tiff]

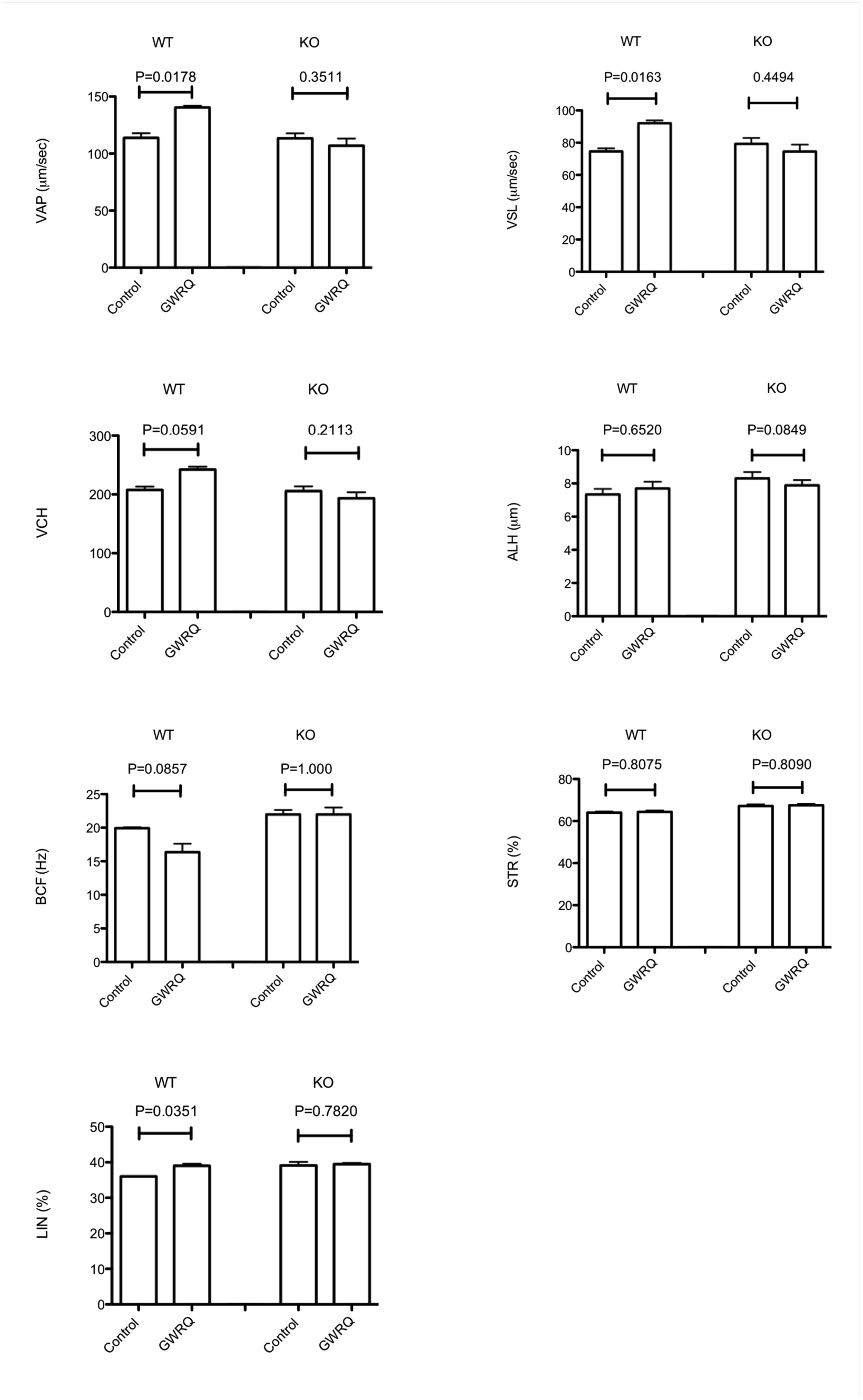

Supplement: Additional file 3 — Figure S3. CASA parameters. Data were collected by analyzing fresh sperm frosignificant (pm wild type (n=10) and trophinin null (n=3) mice. Asterisks represent statistical < 0.05) by unpaired two-tailed t-test. N. S., not significant. [file 1477-7827-10-101-S3.tiff]
